# ErbB activation signatures as potential biomarkers for anti-ErbB3 treatment in HNSCC

**DOI:** 10.1371/journal.pone.0181356

**Published:** 2017-07-19

**Authors:** Diego Alvarado, Gwenda F. Ligon, Jay S. Lillquist, Scott B. Seibel, Gerald Wallweber, Veronique M. Neumeister, David L. Rimm, Gerald McMahon, Theresa M. LaVallee

**Affiliations:** 1 Kolltan Pharmaceuticals., New Haven, Connecticut, United States of America; 2 Monogram Biosciences, Laboratory Corporation of America® Holdings, South San Francisco, California, United States of America; 3 Yale Pathology Tissue Services, Yale University, New Haven, Connecticut, United States of America; Technische Universitat Dresden, GERMANY

## Abstract

Head and neck squamous cell carcinoma (HNSCC) accounts for 3–5% of all tumor types and remains an unmet medical need with only two targeted therapies approved to date. ErbB3 (HER3), the kinase-impaired member of the EGFR/ErbB family, has been implicated as a disease driver in a number of solid tumors, including a subset of HNSCC. Here we show that the molecular components required for ErbB3 activation, including its ligand neuregulin-1 (NRG1), are highly prevalent in HNSCC and that HER2, but not EGFR, is the major activating ErbB3 kinase partner. We demonstrate that cetuximab treatment primarily inhibits the ERK signaling pathway and KTN3379, an anti-ErbB3 monoclonal antibody, inhibits the AKT signaling pathway, and that dual ErbB receptor inhibition results in enhanced anti-tumor activity in HNSCC models. Surprisingly, we found that while NRG1 is required for ErbB3 activation, it was not sufficient to fully predict for KTN3379 activity. An evaluation of HNSCC patient samples demonstrated that NRG1 expression was significantly associated with expression of the EGFR ligands amphiregulin (AREG) and transforming growth factor α (TGFα). Furthermore, NRG1-positive HNSCC cell lines that secreted high levels of AREG and TGFα or contained high levels of EGFR homodimers (H11D) demonstrated a better response to KTN3379. Although ErbB3 and EGFR activation are uncoupled at the receptor level, their respective signaling pathways are linked through co-expression of their respective ligands. We propose that NRG1 expression and EGFR activation signatures may enrich for improved efficacy of anti-ErbB3 therapeutic mAb approaches when combined with EGFR-targeting therapies in HNSCC.

## Introduction

Head and neck squamous cell carcinoma (HNSCC) refers to cancers of squamous cell histology that arise from the paranasal sinuses, nasal cavity, oral cavity, pharynx and larynx and it represents 3–5% of all tumors diagnosed (http://seer.cancer.gov/). Most patients with HNSCC present with Stage III to IVB disease, which is treated aggressively with multimodality therapy. Treatment failure rates remain high with 60% and 30% of patients having local and distant treatment failure, respectively [1]. Among patients who develop recurrent/metastatic disease, survival is poor with median survival generally less than 1 year and treatment options are limited. HNSCC remains a major medical issue with only two targeted therapies approved to date, the chimeric anti-EGFR monoclonal antibody (mAb) cetuximab and recently anti-PD-1 mAbs.

The ErbB/HER family of receptor tyrosine kinases (RTKs) drives the growth of several solid tumor types through overexpression, mutation, or ligand-mediated signaling [2,3]. EGFR signaling promotes tumor growth and progression. Cetuximab, an inhibitory EGFR mAb has received regulatory approval for the treatment of patients with locally or regionally advanced HNSCC in combination with radiation therapy and in combination with platinum/5-FU in patients with recurrent/metastatic HNSCC. Cetuximab is also approved for treatment as a single agent in patients with recurrent/metastatic HNSCC who have had tumor progression following platinum-based therapy. However, no standard of care exists for patients with recurrent /metastatic disease who have progressed during or after initial systemic treatment. A need remains to identify rational combinations for treatment of HNSCC to improve the survival of patients.

ErbB3 (HER3), the kinase-impaired member of the family, functions as an obligate heterodimer with the other kinase-active members of the ErbB family [4,5]. ErbB3 activation in normal tissue and in a subset of solid tumor types is regulated by the production or availability of the neuregulin (NRG) family of ligands [6–8]. In tumors that highly overexpress HER2 through gene amplification, ErbB3 can also be activated directly by HER2 through mass action in a ligand-independent fashion [9–11]. Irrespective of the activation source, phosphorylated ErbB3 strongly couples to the PI3K/AKT pathway by means of six p85-binding sites and elicits a potent anti-apoptotic signal that drives tumor growth and survival [2,12,13]. Increased ErbB3 activation and signaling in response to EGFR, HER2, BRAF/MEK, PI3K/mTOR pathway inhibitors and several chemotherapies has been identified as a mechanism of resistance to a wide array of approved targeted therapies [14–17]. While NRG1 expression has been reported to be a biomarker of response to ErbB3 targetings mAbs, its use alone may be insufficient [18]. Thus, optimizing anti-ErbB3 treatment by selecting an enriched patient population and the right therapeutic combination is needed.

Dual targeting of ErbB receptors with mAbs or small molecules kinase inhibitors has proven to be a successful strategy to fully inhibit ErbB receptor signaling activity in several cancer indications. Combination of the HER2 antibodies trastuzumab and pertuzumab, each targeting a different epitope in HER2, resulted in a striking improvement in patient overall survival in a phase 3 trial of HER2-amplified metastatic breast cancer [19,20]. Similarly, combination of the EGFR-targeting agents cetuximab and afatinib in lung cancer has resulted in early signs of clinical benefit in patients that progressed on erlotinib [21]. The underlying mechanism for drug synergy depends on the target and the clinical context, and may involve full inhibition of an otherwise partially engaged target, or reversing acquired resistance to a targeted therapy. Given these published results, and the known role of ErbB3 as a potent pro-tumorigenic receptor, combination strategies using ErbB3 inhibitors and other approved ErbB-targeting therapies might be expected to yield improved clinical benefit compared to current approved ErbB therapies.

We show that NRG1 is most highly overexpressed in HNSCC relative to all solid tumors evaluated. As cetuximab is an approved treatment in this indication, evaluating combination treatment with cetuximab and anti-ErbB3 mAbs is warranted. However this strategy may not be generally applied to cetuximab as NRG1 expression was markedly lower in colorectal cancer (CRC), another tumor type where cetuximab is an approved therapy and where EGFR signaling has been implicated.

Here, we explore the anti-tumor activity of KTN3379 (also known as CDX-3379) in HNSCC and evaluate dual ErbB3/EGFR blockade with KTN3379 and cetuximab. Previously, preclinical studies using KTN3379 have demonstrated single agent anti-tumor activity and enhancement of cetuximab activity in a model of HNSCC [22]. We sought to understand which factors would influence the ability of ErbB3-targeting antibodies to show anti-tumor properties in the potential treatment of HNSCC using KTN3379. KTN3379 is a human IgG1 monoclonal antibody that potently inhibits ErbB3 signaling when activated by NRG1 or, separately, in a ligand-independent manner through HER2 overexpression. KTN3379 binds to a unique ErbB3 epitope in domain 3 and the hinge region of domain 2, and locks ErbB3 in its auto-inhibited state, preventing its activation through either mechanism [23]. In addition, KTN3379 has been engineered for increased serum exposure by virtue of three amino acid substitutions in the Fc region (M252Y/254T/T256E; YTE) that enhance FcRN binding [24]. KTN3379 is currently in clinical trials for patients with various solid tumors.

We show that ErbB3 inhibition by KTN3379 inhibits mainly AKT signaling while cetuximab predominantly attenuates the ERK pathway, providing a mechanistic rationale for the observed enhanced anti-tumor activity of KTN3379 when combined with cetuximab. Furthermore, we provide evidence that HER2, and not EGFR, is the major protein kinase responsible for activating ErbB3 in HNSCC. Lastly, an analysis of patient tumor samples reveals an association between RNA expression levels of NRG1, and two EGFR ligands amphiregulin (AREG) and transforming growth factor α (TGFα). Higher levels of AREG and TGFα correlate with more potent KTN3379 activity, as do high levels of EGFR homodimers, a surrogate for ligand-receptor interaction. Together, our data support the clinical evaluation of KTN3379 in HNSCC and provides a scientific rationale that supports the combination of EGFR and ErbB3-targeting mAbs for the treatment of this tumor type. In addition, we suggest new potential tumor-specific biomarkers that may improve on using NRG1 alone and allow for selection of a subset of HNSCC patients for the combination of KTN3379 with EGFR inhibitors.

## Materials and methods

### Cell lines

FaDu, Cal27, Detroit562, and SCC9 cells were purchased from ATCC and grown as recommended by the distributor. UNC7, UNC10, SCC61 and SCC35 cells were kind gifts from Dr. Natalia Issaeva and Dr. Wendell Yarbrough (Yale Medical School, New Haven, CT). UNC10 and UNC7 cells were grown in MEM, and supplemented with non-essential amino acids (NEAA), 5 μg/mL of insulin/transferrin and 5 ng/mL of sodium selenite. SCC35 and SCC61 were grown in DMEM/F12 (1:1) and supplemented with 400 ng/mL of hydrocortisone. Detroit562 cells were grown in MEM and supplemented with NEAA and sodium pyruvate. Cal27 cells were grown in RPMI. FaDu cells were grown in MEM. SCC9 cells were grown DMEM/F12 (1:1) and supplemented with 15 mM HEPES, 500 μM sodium pyruvate and 400 ng/mL hydrocortisone. Cells were obtained in 2013, with the exception of SCC9 which was obtained in 2015. All cell lines were grown at 37°C with 5% CO_2_ and supplemented with 10% FBS, GlutaMAX and Pen Strep (Thermo Fisher Scientific)

### Antibody purification

KTN3379, cetuximab and pertuzumab were cloned, expressed and purified in-house as described below. Plasmids encoding the light and heavy chain of each antibody were transiently co-expressed in Expi293 cells (Thermo Fisher Scientific) using polyethylenimine. Conditioned media containing the antibodies was flowed over MabSelect SuRe Protein A (GE Healthcare). Bound antibody was washed with PBS and eluted in 0.1 M glycine pH 2.7 and immediately neutralized with 1 M Tris pH 7.4. Purified antibody was buffer exchanged into PBS by tangential flow filtration using a Sartorius Slice ECO with a 30 KDa cutoff Hydrosart^®^ cassette. Antibodies were purified with low endotoxin (<1 EU/mg), and were >95% pure and >95% monomeric as assessed by SDS-PAGE, and ultra-high pressure size exclusion chromatography (SEC-UPLC) using a UPLC BEH 200 column in a Waters Acquity instrument.

### Proliferation assays

Cells were seeded on black 96-well plates at a density of 1,000–3,000 cells/well and allowed to attach overnight. Antibody titrations were carried out in media containing reduced fetal bovine serum (FBS). Cells were allowed to grow between 3 and 7 days, until control-treated cells reached near-confluence. Cell growth was monitored indirectly using CellTiter-Glo (Promega) as described by the manufacturer, and luminescent data were read in a Synergy HT plate reader (BioTek). Data were normalized to control-treated samples, plotted as a function of the log-transformed drug concentration, and fit to a 4-parameter non-linear regression function using GraphPad Prism. KTN3379 activity was calculated by comparing the maximal anti-proliferative effect in the KTN3379 + cetuximab arm versus the cetuximab alone arm. Statistical significance was determined using a Student’s t-test, with a p-value cutoff of <0.05.

### Western blot analysis

Cells were plated in 6-well plates in complete media, and allowed to attach overnight in reduced serum. Cells were treated with 100 nM of each antibody for 2 hours at 37°C, washed once with phosphate buffer saline (PBS) and lysed in a buffer containing 25 mM Tris pH 7.4, 150 mM NaCl, 1% Triton X-100, 1 mM EDTA, 1 mM sodium orthovanadate and complete protease inhibitors (Roche). Insoluble material was cleared by centrifugation and the remaining sample was diluted in Laemmli sample buffer (Boston BioProducts). Samples were subjected to SDS-PAGE, transferred to nitrocellulose, and blocked in 5% nonfat dry milk in TBST (TBS + 0.1% Triton X-100) for 1 hour at room temperature. Antibodies utilized in these studies were purchased from Cell Signaling and used as recommended by the manufacturer. Primary antibody incubations were done overnight at 4°C. Washed membranes were incubated with horseradish-peroxidase (HRP)-conjugated secondary antibodies for 1 hour at room temperature, washed again, and incubated with SuperSignal West Pico Chemiluminescent Substrate (BioRad). Images were captured in a ChemiDoc XRS+ Imager (BioRad).

### Ligand secretion assays

Cells were plated in 6-well plates in complete medium, allowed to attach overnight, and treated with 100 nM cetuximab (to block ligand binding to EGFR and its subsequent internalization, and promote ligand accumulation in the media) in media without serum for 48 hours. AREG and TGFα in conditioned media were quantified by ELISA (quantikine, R&D Systems) following manufacturer’s instructions.

### AKT phosphorylation

Phospho-AKT antibody pairs were purchased from R&D Systems. 30,000 cells were plated in complete growth media and allowed to grow for 2 days. Cells were then treated with or without 100 nM KTN3379 in quadruplicate for 2 hours at 37°C. Cells were lysed with a buffer containing 50 mM Tris pH 7.4, 150 mM NaCl, 1% Triton X-100, 0.25% deoxycholate, 1 mM EDTA, 5 mM NaF, 2 mM sodium orthovanadate, and protease inhibitor tablets (Roche). Lysates and phospho-AKT standards (R&D) were added to a Meso Scale Discovery (MSD) 96-well ELISA plate that was coated with a capture AKT antibody and blocked with 1% bovine serum albumin in PBS. Lysates were incubated overnight at 4°C with shaking, and the plates were subsequently washed with PBS with 0.05% Tween-20, and incubated with an AKT detection antibody for 2 hours at room temperature. After washing, plates were incubated with streptavidin-sulfotag (MSD) for 1 hour at room temperature. Plates were washed, incubated with read buffer (MSD) and read in a Meso Scale Discovery QuickPlex SQ 120 reader. KTN3379-mediated inhibition was calculated by obtaining the ratio of phospho-AKT (in pg/mL) in treated versus the control cells.

### Gene expression analysis

TCGA head and neck squamous cell carcinoma mRNASeq gene expression data for NRG1 (probe set 206343_s) (y-axis) and TGFα or AREG (x-axis) were sourced from the Broad Institute’s Firehose and plotted using TIBCO Spotfire. Units on both axes were upper-quartile normalized and log2 transformed RSEM (RNASeq by Expectation Maximization). Linear regression was used to quantify the relationship between genes and p-values were generated using an F-test to determine if the independent variable (X) predicted a significant proportion of the variance of the dependent variable (Y). The p-value is then calculated from the F-distribution where the F-statistic is calculated with the sum of squares between the estimated line and the total mean of the *y*_*i*_'s having one degree of freedom as numerator and the residual sum of squares divided by the number of degrees of freedom (n-2) as denominator (3, 4). Evaluation of NRG1 expression was also done with probe sets 3092808 and 32719_at, yielding similar results. The analysis was performed by Compendia Bioscience (Thermo Fisher Scientific).

### VeraTag^®^ assays

All VeraTag assays were performed on formalin-fixed, paraffin-embedded (FFPE) tissues or cell lines by Monogram Biosciences. FFPE blocks were sectioned at 5 micron thickness and placed on positively charged glass slides. Released fluorescent VeraTags were detected and quantified by capillary electrophoresis (CE). Following the VeraTag assay, the slides were hematoxylin and eosin (H&E) stained, tumor area identified and circled, and the fluorescent signal from the released VeraTag was normalized to sample buffer volume and tumor area to give units of relative fluorescence per square millimeter of tumor (RF/mm^2^). A panel of cell line controls was assayed together with the samples and used to control for batch-to-batch variability and allow for the comparison of samples over time. The following antibody pairs were used: HER2; anti-HER2 mouse mAb e2-4001 paired with biotinylated anti-HER2 mouse mAb 3B5 (Thermo Fisher Scientific). ErbB3; anti-ErbB3 mouse mAb 2B5 (Thermo Fisher Scientific) paired with biotinylated anti-ErbB3 mouse mAb B9A11 (Monogram Biosciences). H11D (EGFR homodimer); equal concentrations of anti-HER1 rabbit mAb D38B1 (Cell Signaling Technology) labeled with either a fluorescent VeraTag reporter or biotin. EGFR; anti-EGFR rabbit mAb D38B1 (Cell Signaling Technology) and goat F(ab’)_2_ anti-mouse IgG (Southern Biotech) labeled with a fluorescent VeraTag reporter via a disulfide bond [25].

### Mouse xenograft studies

Charles River Discovery Services specifically complies with the recommendations of the Guide for Care and Use of Laboratory Animals with respect to restraint, husbandry, surgical procedures, feed and fluid regulation, and veterinary care. The animal care and use program at CR Discovery Services is accredited by the Association for Assessment and Accreditation of Laboratory Animal Care International, which assures compliance with accepted standards for the care and use of laboratory animals. All the procedures related to animal handling, care and the treatment in the study were performed according to the guidelines approved by the Institutional Animal Care and Use Committee (IACUC) of WuXi AppTec following the guidance of the Association for Assessment and Accreditation of Laboratory Animal Care (AAALAC). The animals were daily checked for any effects of tumor growth and treatments on normal behavior such as mobility, food and water consumption, body weight gain/loss, eye/hair matting and any other abnormal effects. Death and observed clinical signs were recorded. Studies performed by Champions Oncology were overseen by Sobran IACUC (Rangos Building, Baltimore, MD 21205).

KTN3379, cetuximab and a control IgG1 antibody were dosed intraperitoneally twice a week at 10 mg/kg, except for the Cal27 model, which was dosed at 20 mg/kg. 5x10^6^ FaDu cells were implanted in athymic nude female mice and the study was conducted by Charles River Laboratories. 1x10^7^ Cal27 cells were implanted in a 1:1 mixture with matrigel and the study was conducted by WuXi AppTec. Patient-derived xenograft CTG0434 tumor fragments were implanted subcutaneously in NCR nude female mice, and the study was conducted by Champions Oncology. OE21 tumor-bearing mice were dosed with KTN3379 and cetuximab at 10 mg/kg as described above, and studies were performed by MedImmune. Percent tumor growth inhibition (TGI) was determined by the formula %TGI = [(C_t_-C_0_)-(T_t_-T_0_)]/(C_t_-C_0_) x 100 where T_t_ = treated arm tumor volume at time t; T_0_ = treated arm tumor volume at the start of dosing; C_t_ = control arm tumor volume at time t; C_0_ = control tumor volume at the start of dosing. All agents were formulated in PBS. 8 animals were dosed per experimental arm in all studies with the exception of the CTG-0776 model, in which 10 animals were dosed per arm. In all studies, all tumors were grown until the mean tumor size reached 100–200 mm^3^ (depending on the model) and animals were sorted to ensure the same starting mean tumor size across all experimental groups. All treatments in these studies were acceptably tolerated. Statistical significance was determined by performing a Student’s t-test using GraphPad Prism, and a p-value cutoff of P<0.05. Figure graphs reflect the mean and the calculated standard error of the mean (S.E.M.) for each experimental group.

### Phospho-ErbB reverse-phase microarray (RPMA)

Cal27 cells were treated with 100 nM of cetuximab, pertuzumab, or KTN3379 for 2 hours at 37°C, washed, and lysed as described for western blots. RPMA was used to measure the effects of antibody treatment on the phosphorylation content of EGFR, HER2, and ErbB3 and were performed by Theranostics Health. Briefly, 6 nL of cell lysates were arrayed onto glass-backed nitrocellulose slides and arrayed in four replicates, along with controls for each target. The printed slides were subjected to immunostaining with fluorescently-tagged antibodies specific to phosphorylated EGFR, HER2, and ErbB3. The expression signal was background subtracted and normalized to total protein, determined by Spyro Ruby Protein stain (Invitrogen).

### Quantitative in situ hybridization (QISH)

Archived FFPE tumor tissue samples were analyzed for expression levels of NRG1β, and ErbB3 using an in situ hybridization (ISH) protocol modified for immunofluorescent staining and quantification as described previously [26]. ISH for these mRNAs was performed using the RNAscope 2.5 LS reagent and assay kit developed by Advanced Cell Diagnostics (ACD) and a Leica automated Bond RX staining platform. The mRNA probes were also purchased from ACD. Briefly, 5 μm thick whole tissue sections were de-paraffinized and treated with heat and protease digestion followed by hybridization with the target probes. For each specimen the house keeping gene *Ubiquitin C (UBC)* was assessed as positive control and the bacterial gene *DapB* as a negative control and to determine threshold to noise in a quantitative manner. The mRNA specific hybridization signals were detected with Cy5-tyramides. Sections were then incubated with 0.3% bovine serum albumin (BSA) in 0.1 M Tris-buffered saline for 20 minutes at room temperature followed by incubation with a wide-spectrum rabbit anti-cow cytokeratin antibody (Z0622 1:100, DAKO Corp, Carpinteria, CA) for 20 minutes at room temperature. The cytokeratin signal was detected with Alexa 546 conjugated goat anti-rabbit (1:100, Molecular Probes), incubated for 20 minutes at room temperature. All staining procedures were standardized and automated on the Bond RX staining platform. Slides were then mounted using ProlongGold plus 4,6-diamino-2-phynylindole (DAPI). Quantification of the mRNAs of interest was performed using the method of automated quantitative analysis as described previously [27].

#### Automated quantitative analysis (AQUA)

AQUA^®^ is a method to objectively and accurately measure biomarker expression within defined tumor areas and subcellular compartments based on co-localization with cytokeratin and DAPI. Multiplexed staining of a target of interest and cytokeratin allows to define the tumor area (in case of epithelial tumors) and to measure the pixel intensity of the target within the cytokeratin positive cells. After immunofluorescent staining/ISH of the tissue, a series of monochromatic, high-resolution images were captured using the PM2000 image workstation. For each field of view (FOV) on a whole tissue section images for three different channels were obtained. Signal from DAPI stain visualizes the nuclei, cytokeratin is visualized with Alexa 546 and the mRNA of interest with Cy5. The cytokeratin signal creates a tumor mask, which allowed the measurement of pixels of the target of interest within this defined tumor area and/or within subcellular compartments.

#### Assay/probe validation and quality control

The probes and assays were validated using mRNA specific index tissue micro arrays (TMAs) consisting of probe specific positive and negative FFPE cell line pellets. These index TMAs were used to test specificity of the probes, the performance and reproducibility of the assay and were run alongside tumor sample testing for quality control purposes. Each sample was stained for the mRNAs of interest, for UBC and dapB. Only tumor samples with sufficient UBC expression levels were further included in the analysis. DapB quantification allows measurement of unspecific noise and background levels and was used to calculate tumor specific signal to noise ratio for each probe of interest.

## Results

### High prevalence of NRG1 expression measured in human HNSCC

**Fig 1:**
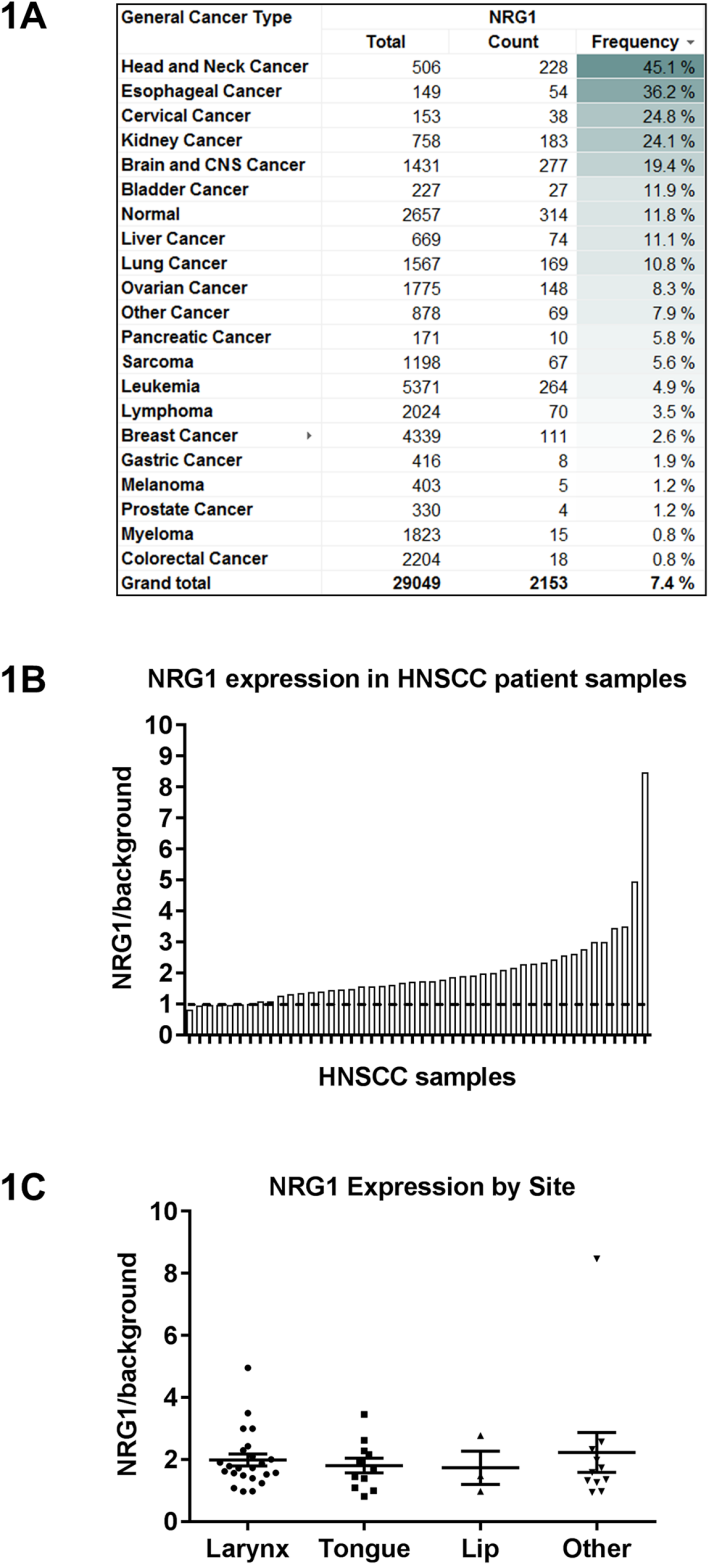
Prevalence of NRG1 expression in HNSCC and other tumor types. **(A)** NRG1 RNA overexpression was found to be most prevalent in HNSCC compared to other major tumor types, and overexpressed in nearly half of all head and neck tumors examined. RNA overexpression is defined as > 4-fold expression over the median expression across all samples (> 29,000). By contrast, NRG1 was overexpressed only in ~ 1% of colorectal cancer. TCGA data were used for this analysis. **(B and C)** A quantitative ISH-based assay demonstrates detectable NRG1 expression in the majority of human HNSCC tumors **(B)** and is independent of the histological site **(C)**. Values reflect the ratio of the NRG1 probe signal over that of a control probe.

ErbB3 activation is driven by the neuregulin family of ligands (NRG1 and NRG2, specifically) in a subset of solid cancers [23,28]. We found that HNSCC expressed the highest levels of NRG1 compared to all major tumor types (>29,000 samples) in agreement with previously published studies [29]. TCGA data analysis revealed that NRG1 is overexpressed in HNSCC with a prevalence of 45% (N = 228 tumor samples; Fig 1A), where overexpression is defined as a 4-fold increase in mRNA levels over the median value across all samples (tumor samples and normal tissue). Similarly, NRG1 overexpression was also observed in tongue and oral cavity tumors, relative to normal tongue and oral cavity tissue (unpublished observations). By contrast, NRG1 was overexpressed in only 1% of colorectal cancers, a tumor type where anti-EGFR monoclonal antibodies (cetuximab and panitumumab) are currently approved for use. In addition, we carried out a subset analysis of NRG1 expression in HNSCC according to the two most prevalent molecular subtypes: HPV and PI3K status. In HPV-negative tumors, which accounts for ~80% of all HNSCC [30], NRG1 was more highly expressed than in HPV-positive tumors (p-value of 4.95E-8) (S1 Fig). Similarly, NRG1 was more highly expressed in HNSCC tumors bearing wild-type PI3K, with a prevalence of ~85% of HNSCC [31] compared to PI3K-mutated tumors (defined by known activating mutations in exons 9 and 20) (p-value of 0.0013) (S1 Fig). Nevertheless, NRG1 expression in both HPV-positive and PI3K-mutated subsets was still high relative to most other tumor types). We further investigated NRG1 expression in two HNSCC tumor microarrays from 46 patient samples using a quantitative RNA-based in-situ hybridization assay (QISH) with a probe that recognizes the NRG1β isoform [32]. NRG1 was detectably expressed in 85% of the samples (39/46) relative to a negative control, suggesting that most HNSCC tumors express NRG1, as previously reported [33] (Fig 1B and 1C). The presence of the NRG1 ligand suggests that ErbB3-targeting inhibitors, such as KTN3379, may be useful in a large subset of HNSCC where NRG1 expression leads to ErbB3 signaling.

### ErbB3 and ErbB co-receptors are widely expressed in HNSCC patient samples

**Fig 2:**
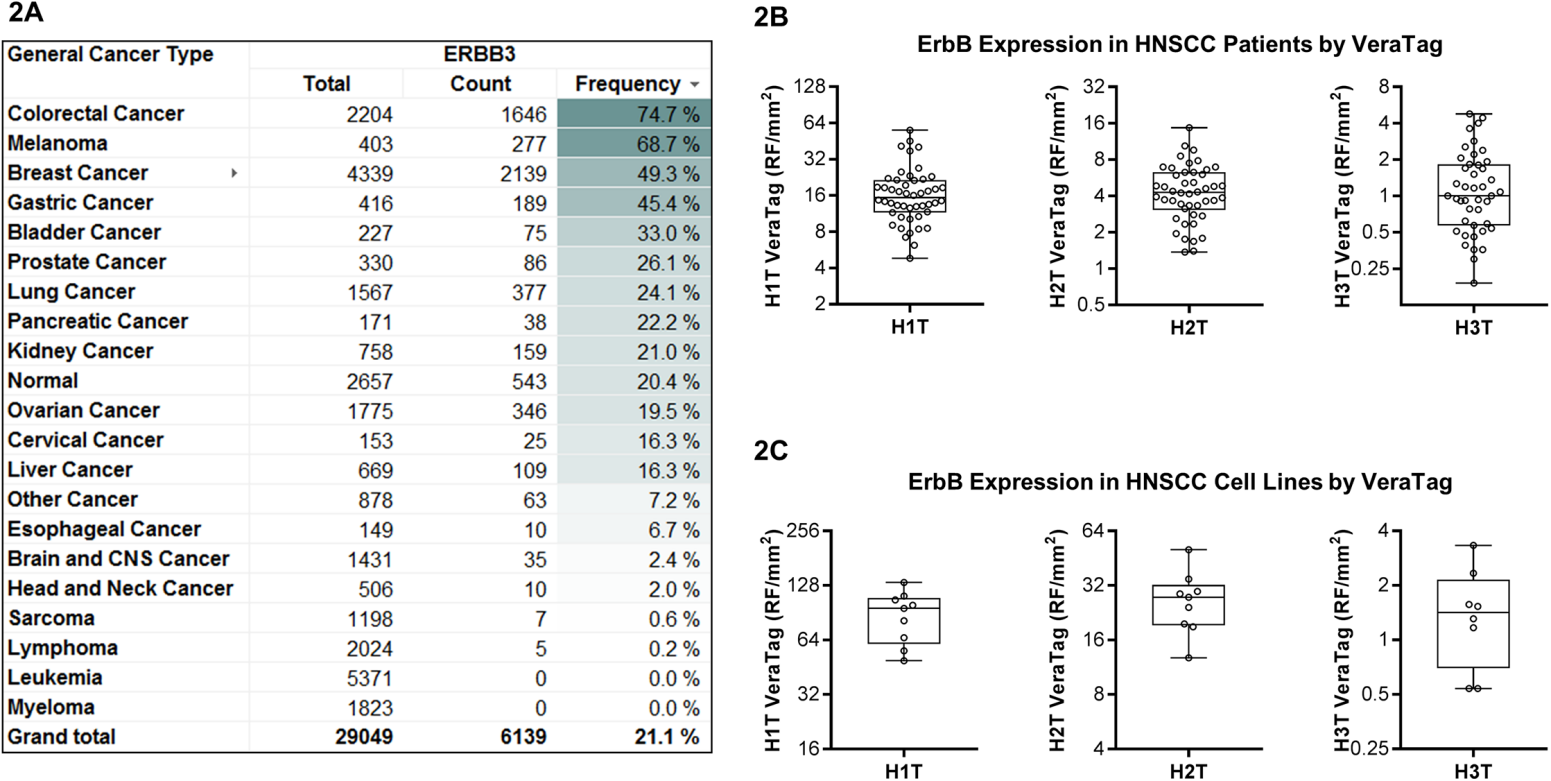
Prevalence of EGFR, HER2, and ErbB3 expression in HNSCC. **(A)** ErbB3 is widely expressed, but not overexpressed, in HNSCC. RNA overexpression was defined as > 4-fold expression over the median expression across all tumor types. TCGA data were used for this analysis **(B)** ErbB receptors were expressed in the majority of HNSCC patient tumor samples (n = 46), as determined by VeraTag proximity-based immunoassays. Total EGFR (H1T), HER2 (H2T), and ErbB3 (H3T) levels are shown **(C)** The same pattern of ErbB protein expression was observed in a panel of 8 HNSCC cell lines.

Since NRG1 is expressed in the majority of HNSCC tumors, we asked whether ErbB3 itself is also expressed in HNSCC patient samples and thus, may serve as a tumor target for anticancer therapy. We found that unlike NRG1, ErbB3 expression in HNSCC is highly prevalent but rarely overexpressed relative to other tumor types (Fig 2A, S2 and S3 Figs), as determined by mRNA analysis. Interestingly, we also observed that ErbB3 was also rarely overexpressed in squamous lung cancer samples (unpublished observations), suggesting that ErbB3 signaling in cancers of squamous origin, which often express NRG1, may lead to downregulation of ErbB3 mRNA expression [34]. Nevertheless, we found that the majority of HNSCC tumors express ErbB3 at the protein level. Using the VeraTag proximity-based immunoassay [35,36] we observed widespread ErbB3 protein (H3T) expression in the same patient tumor cohort analyzed for NRG1 (Fig 2B). Using the H3T VeraTag assay, we also detected ErbB3 protein in the majority of HNSCC cell lines examined (Fig 2C, S2 Fig). Cell surface expression analysis using flow cytometry confirmed the ErbB3 expression pattern observed by H3T VeraTag in the same cell lines (S2 Fig).

Given that ErbB3 activation requires both NRG1 and another ErbB co-receptor, we also measured protein expression of EGFR and HER2 in the HNSCC cell lines and patient cohort. Consistent with previous reports [37], EGFR was highly expressed in HNSCC patient samples and cell lines, and HER2 expression was highly prevalent and present at moderate levels (Fig 2C, S2 and S3 Figs). Thus, all the factors required for ErbB3 activation (ligand, receptor and potential ErbB co-receptors) were expressed in a large subset of HNSCC samples tested.

### NRG1 positivity is necessary but insufficient to predict KTN3379 activity in HNSCC

**Fig 3:**
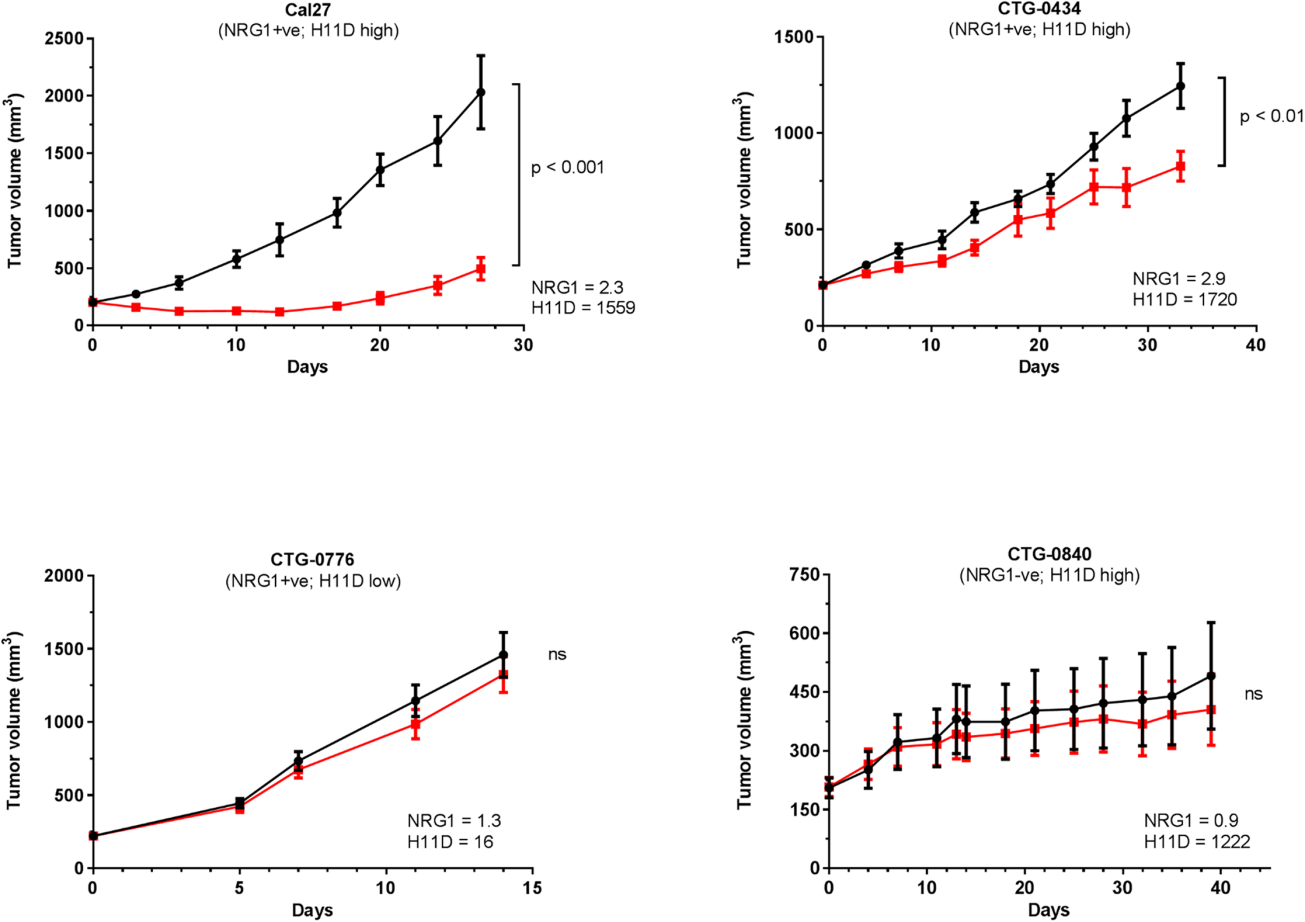
KTN3379 demonstrates single agent *in vivo* anti-tumor activity in some, but not all NRG1-positive HNSCC models. Three patient derived xenograft (PDX) models of HNSCC and one cell line (Cal27) were evaluated for expression of NRG1 and EGFR homodimer (H11D) levels. Single agent KTN3379 dosing resulted in significant anti-tumor efficacy in two NRG1 positive models (Cal27 and CTG-0434), but had no effect in NRG1-positive model (CTG-0776) and one NRG-negative model (CTG-0840). Evaluation of EGFR homodimer (H11D) levels indicated that KTN3379 was efficacious in models that are both NRG1-positive and express high H11D levels, but not in models where H11D levels are low.

To evaluate the role of NRG1 as a predictive biomarker for KTN3379 response in HNSCC, we evaluated KTN3379 *in vivo* anti-tumor activity in a panel of NRG1-positive and NRG1-negative xenograft models (Fig 3), where NRG1 expression was determined by QISH. We found that two NRG1 positive models (Cal27 and CTG-0434) exhibited an anti-tumor response to KTN3379 treatment, while a NRG1 negative model (CTG-0840) did not. Interestingly, KTN3379 did not result in anti-tumor activity in a NRG1-positive model (CTG-0776), suggesting that NRG1 positivity alone may not be sufficient to predict KTN3379 activity in HNSCC.

Although the activity of KTN3379 as a single agent in responsive models was statistically significant, no tumor regressions were observed. This finding was not unexpected, since signaling via other ErbB receptors such as EGFR can compensate for targeted inhibition of ErbB3, suggesting that dual EGFR and ErbB3 blockade may result in improved anti-tumor activity.

### ErbB3 inhibition by KTN3379 enhances the anti-tumor activity of cetuximab *in vitro* and *in vivo*

**Fig 4:**
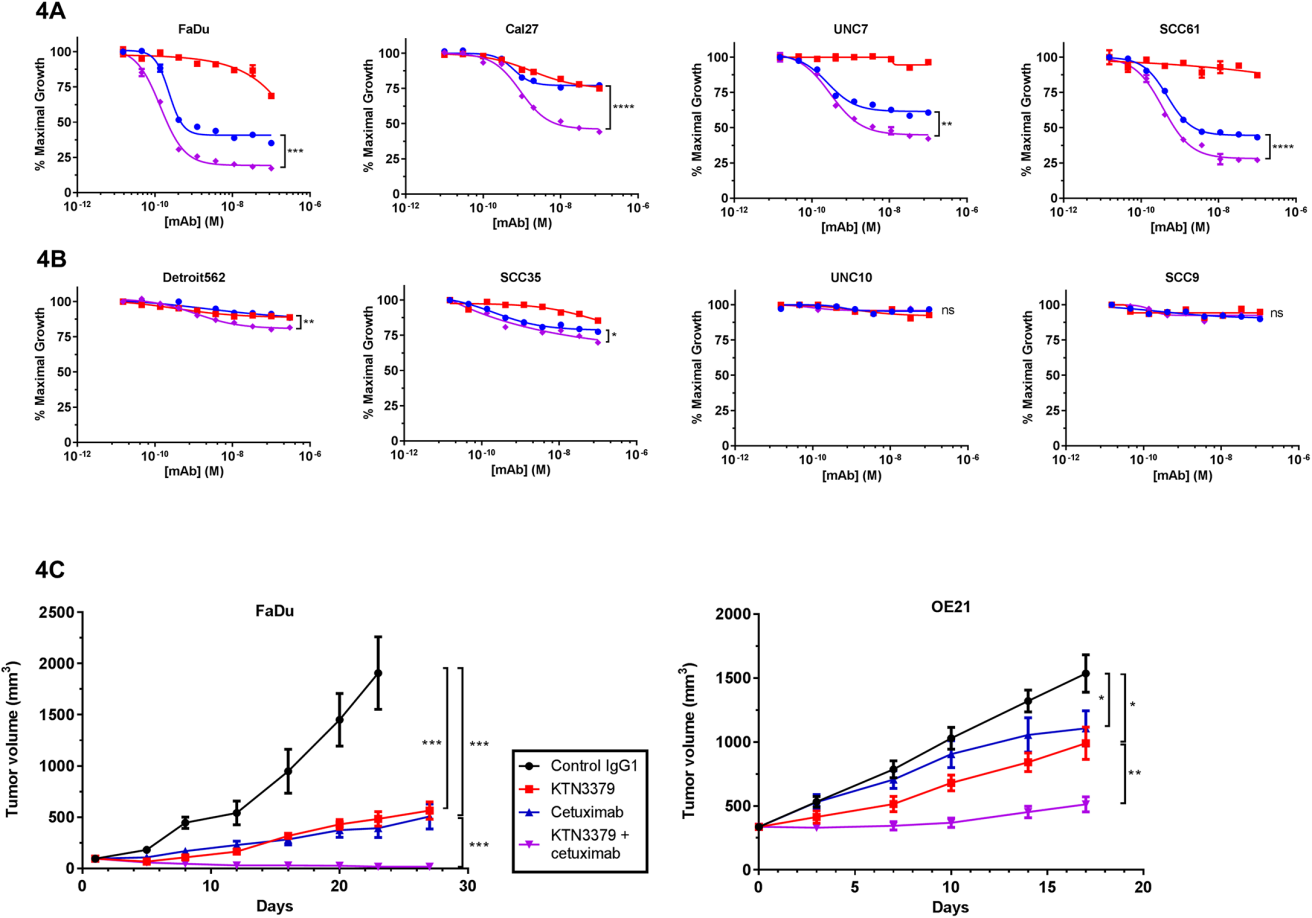
KTN3379 enhances cetuximab anti-proliferative activity *in vitro* and in tumor xenograft models of HNSCC. **(A)** Titration of KTN3379 (red) enhanced cetuximab (blue) anti-proliferative activity in 4 of 8 HNSCC cell lines (top row) and showed modest enhancement of cetuximab-treated activity in a subset of cell lines (Detroit562 and SCC35). In contrast, neither cetuximab nor KTN3379 showed anti-proliferative activity in other HNSCC lines (UNC10 and SCC9). Combination of KTN3379 and cetuximab is shown in purple. **(B)** KTN3379 demonstrated significant single agent activity in 2 HNSCC xenograft models (FaDu and OE21), and enhanced the anti-tumor activity of cetuximab. Animals were dosed intraperitoneally twice weekly at 10 mg/kg. Asterisks denote statistical significance; * p-value <0.05; ** p-value <0.01; *** p-value <0.001; **** p-value <0.0001; ns = not significant.

Clinical and preclinical studies have demonstrated that dual ErbB targeting leads to enhanced efficacy [19,20]. Since ErbB3 is likely activated in many HNSCC tumors, we asked whether ErbB3 inhibition enhances the anti-proliferative effect of cetuximab treatment in HNSCC cell lines. Similar to our observations *in vivo*, ErbB3 inhibition alone with KTN3379 led to modest anti-proliferative activity. Cetuximab treatment resulted in more robust (but incomplete) anti-proliferative activity in 4 HNSCC cell lines (FaDu, Cal27, SCC61 and UNC7), had a modest effect in 2 HNSCC cell lines (Detroit562 and SCC35), and had no effect in 2 others (UNC10 and SCC9) (Fig 4A). Addition of both cetuximab and KTN3379 resulted in significantly increased growth inhibition in cetuximab-sensitive lines compared to each agent alone, suggesting that dual ErbB blockade results in improvedanti-tumor activity. The effects seen *in vitro* were extended *in vivo* using two HNSCC xenograft models, FaDu and OE21 (Fig 4B). Twice a week dosing of KTN3379 at 10 mg/kg resulted in 79% tumor growth inhibition (TGI) in the FaDu model and 45% TGI in the OE21 model. Cetuximab administration using the same dose and schedule led to a TGI of 84% and 36% in the FaDu and OE21 models, respectively. Importantly, combination of both agents led significantly enhanced anti-tumor activity in both models relative to each agent alone, with TGI values of 104% and 85% in the FaDu and OE21 models, respectively. Together, these data indicate that targeting both ErbB3 and EGFR may result in improved responses and patient outcome in HNSCC.

### KTN3379 and cetuximab favor inhibition of independent signaling pathways

We sought to further explore ERK and AKT signaling underlying the enhanced activity observed between cetuximab and KTN3379. Treatment of sensitive cells with 100 nM of cetuximab inhibited the ERK/MAPK signaling pathway (measured by ERK phosphorylation; phospho-ERK), and had a relatively modest effect on AKT phosphorylation (phospho-AKT; S4 Fig). In contrast, KTN3379 treatment inhibited AKT phosphorylation and only modestly decreased phospho-ERK levels. Robust reduction in both ERK and AKT phosphorylation could only be achieved upon addition of both KTN3379 and cetuximab. These data suggest that dual pathway inhibition requires combined shutdown of both ErbB3 and EGFR signaling, and provide an explanation for the enhanced anti-tumor activity of the combination.

In this regard, we measured the inhibition of phospho-AKT elicited by KTN3379 treatment relative to a control antibody (S5 Fig). The data showed that there was a significant correlation between the magnitude of phospho-AKT inhibition by KTN3379 with the anti-proliferative activity of KTN3379 when combined with cetuximab. Additionally, ErbB3 phosphorylation, as measured by VeraTag, was reduced to basal levels after KTN3379 treatment, which also correlated with phospho-AKT inhibition, indicating that ErbB3 phosphorylation may be a useful pharmacodynamic biomarker for clinical studies (S5 Fig).

### ErbB3 activation depends on HER2, but not EGFR

**Fig 5:**
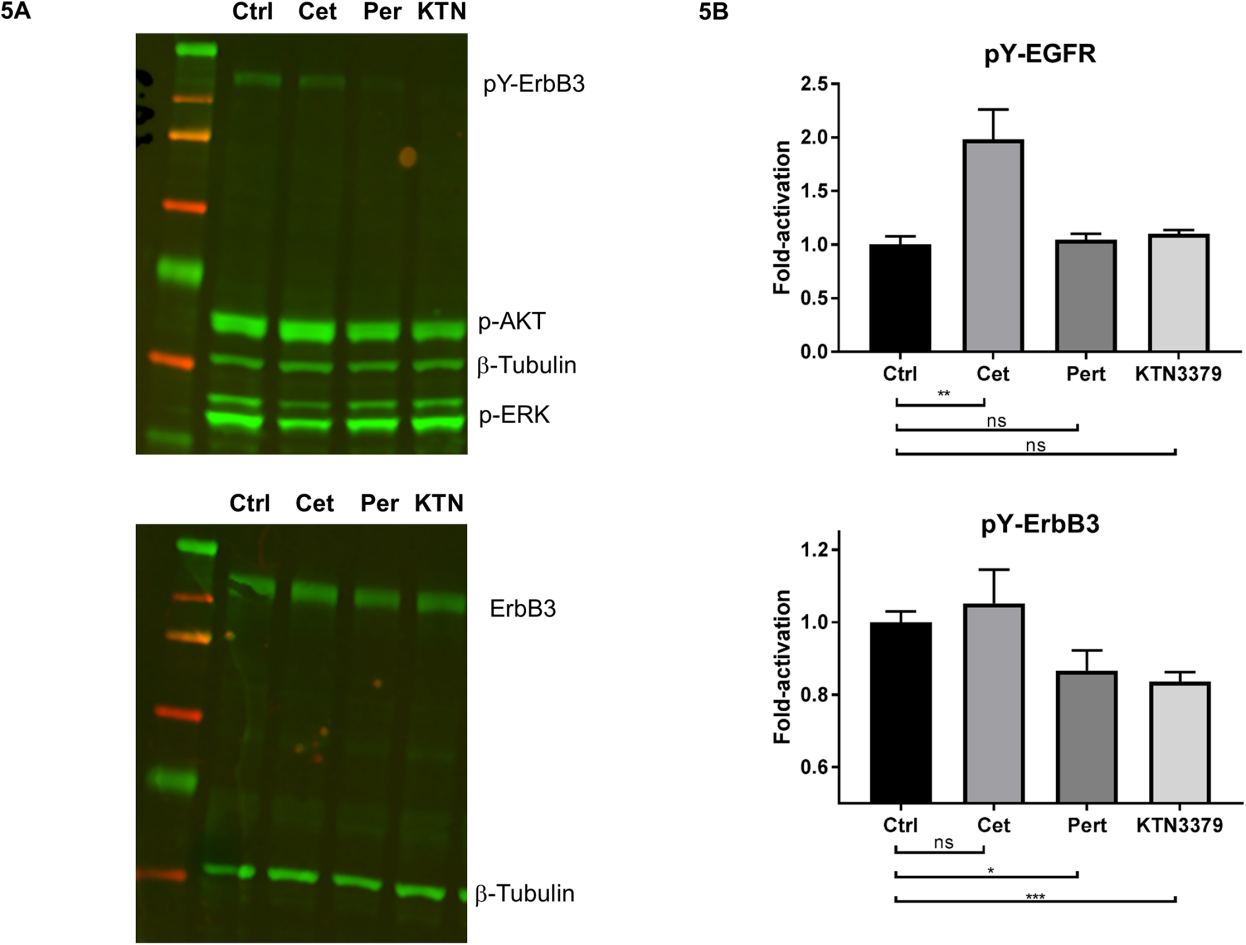
ErbB3 activation depends on HER2. **(A)** Cal27 cells were treated with 100 nM anti-ErbB antibodies and cell lysates were analyzed for ErbB3, AKT and ERK phosphorylation. Addition of KTN3379 (KTN) or pertuzumab (Per) inhibited ErbB3 phosphorylation implicating the role of HER2 as the activating co-receptor for ErbB3. In contrast, cetuximab (Cet) did not inhibit ErbB3 phosphorylation on its own. **(B)** Cal27 cells were treated as described in (A) and phospho-ErbB levels were quantified by RPMA (Reverse-Phase Microarray Assay), yielding results consistent with (A). In addition, cetuximab treatment promoted mild stimulation of EGFR phosphorylation, consistent with previously published results.

Originally, we sought to test a hypothesis that implicated the formation of an EGFR and ErbB3 heterodimer resulting in ErbB3 activation. In this case, cetuximab would be expected to disrupt EGFR and ErbB3 heterodimers leading to a reduction in ErbB3 activity. However, we observed enhanced anti-tumor activity when both KTN3379 and cetuximab were combined suggesting that ErbB3 and EGFR act independently of each other at the receptor level. We sought to identify the target responsible for ErbB3 activation and assessed the effects of several different blocking antibodies on ErbB phosphorylation (Fig 5 and S4 Fig). In order to avoid potential complications arising from receptor dimers enforced by adding high levels of exogenous ErbB ligands, we investigated the effect of individual ErbB-targeting antibodies on the basal phosphorylation of each receptor resulting from autocrine ligand production in cells. Treatment of cetuximab alone in the absence of exogenous EGFR ligands had no effect on ErbB3 phosphorylation and slightly increased the levels of EGFR phosphorylation, consistent with previously described activity as a mild EGFR agonist [38,39]. As expected, KTN3379 inhibited ErbB3 phosphorylation and had no effect on EGFR phosphorylation. These data were extended using a reverse-phase microarray assay from drug antibody-treated cell lysates (Fig 5B). Treatment with the HER2 antagonistic antibody pertuzumab decreased ErbB3 phosphorylation levels, consistent with its known role in preventing formation of HER2-ErbB3 heterodimers. These results suggest that HER2, and not EGFR, is the major kinase responsible for activating ErbB3 in HNSCC. Thus, ErbB3 activation depends on the expression of HER2, ErbB3 and NRG1, all of which are expressed in the majority of HNSCC tumors (Figs 1 and 2).

### KTN3379 anti-tumor activity is associated with high levels of either TGFα or amphiregulin in addition to NRG1 expression

**Fig 6:**
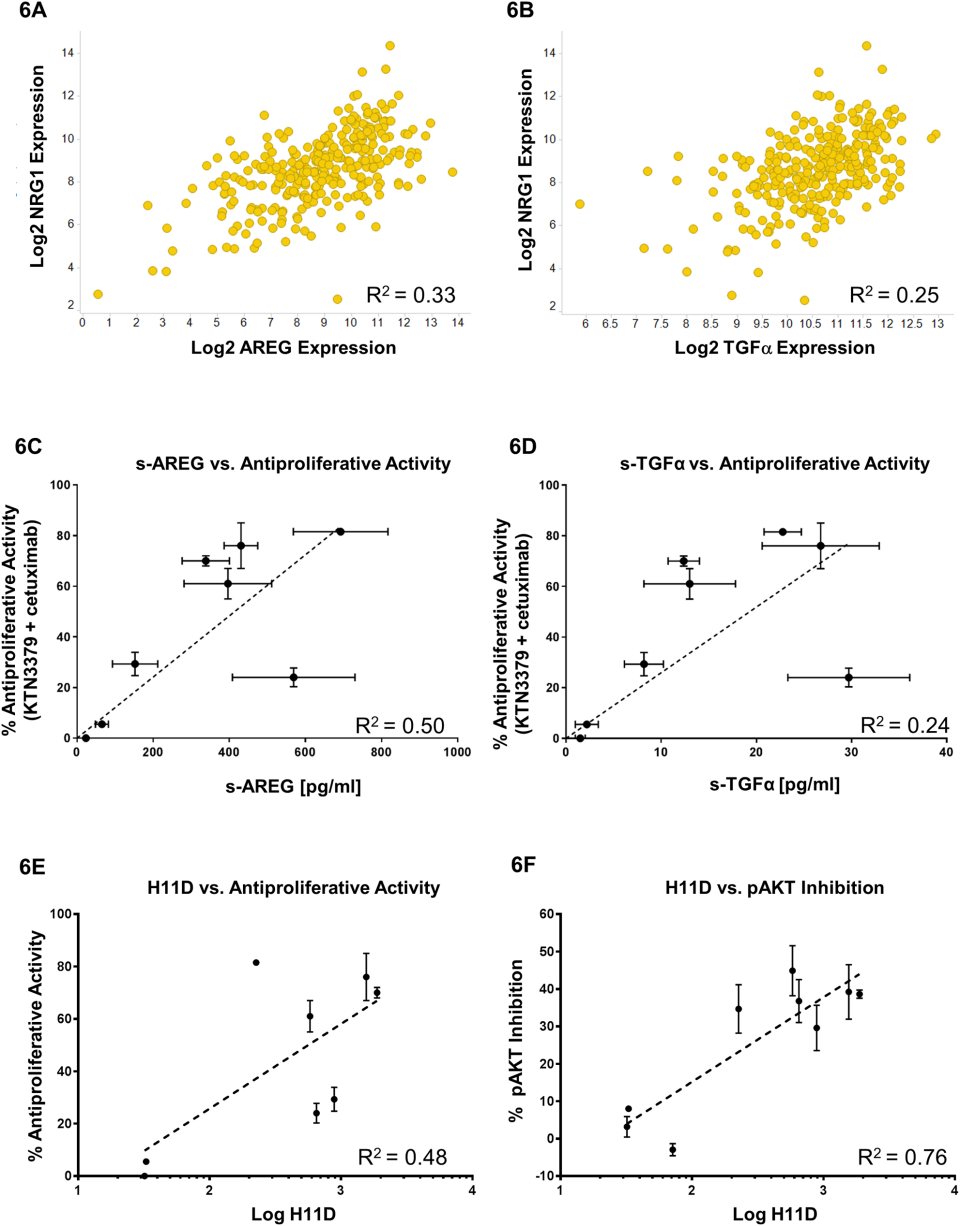
Association of NRG1 expression with AREG and TGFα correlate with KTN3379 activity. **(A and B)** A search of 303 HNSCC patient samples revealed a significant association between NRG1 expression and expression of EGFR ligands AREG (R^2^ = 0.33) and TGFα (R^2^ = 0.25). However, no association was found between NRG1 expression and any of the EGFR ligands in colorectal cancer patient samples (S7 Fig). **(C and D)** High levels of secreted AREG or TGFα protein were found associated with improved KTN3379 activity in HNSCC cell lines. Levels of secreted AREG and TGFα derived from a panel of 8 serum-starved HNSCC cell lines were measured after 48 hours. Supernatant-derived ligand concentrations (in pg/mL) are plotted as a function of the combined anti-proliferative activity of KTN3379 with cetuximab. A linear regression fit of the data gave R^2^ values of 0.50 and 0.24 for AREG and TGFα, respectively. A similar result was observed when ligand concentration data were plotted against KTN3379-mediated phospho-AKT inhibition (S8 Fig). **(E and F)** High levels of EGFR homodimers (H11D) are associated with KTN33379 activity. EGFR homodimer (H11D) levels were evaluated in 8 HNSCC cell lines using a VeraTag immunoassay. H11D values were log-transformed and plotted against the combined anti-proliferative activity of KTN3379 with cetuximab (E), or KTN3379-mediated phospho-AKT inhibition in serum-grown cells (F). Linear regression analysis demonstrates a significant correlation between H11D levels and anti-proliferative activity (R^2^ = 0.48) or phospho-AKT inhibition (R^2^ = 0.76).

All other HNSCC cell lines we have evaluated express detectable levels of NRG1 (S6 Fig). Similar to what we observed in our xenograft studies, a subset of NRG1-expressing models did not respond to KTN3379 treatment as measured by its ability to enhance cetuximab anti-proliferative activity (Fig 4A) or promote phospho-AKT inhibition (S5 Fig). This suggests that NRG1 alone may not be sufficient to predict responsiveness to ErbB3 blockade, even though it has been previously described as a biomarker for enrichment for anti-ErbB3 treatment [8,40]. We performed a search to determine whether there may be a correlation between expression of NRG1 with any of the EGFR ligands that would result in simultaneous activation of both receptors. A query of 303 HNSCC patient samples demonstrated that there was a significant association between NRG1 RNA expression and the expression of AREG and TGFα (Fig 6A and 6B). In contrast, EGFR ligands EGF, betacellulin, HB-EGF, epigen and epiregulin did not show a significant correlation with NRG1 expression (S6 Fig). This finding was not common to all tumor types, since a survey of 537 patient samples of colorectal cancer failed to show a significant association between NRG1 and any EGFR ligands (S7 Fig).

These data established a functional link between EGFR and ErbB3 activity by associating expression of their respective ligands, and suggested that combination of NRG1 with AREG or TGFα may further enrich for KTN3379 anti-tumor activity, particularly when used in combination with cetuximab. To further evaluate this relationship, we measured the levels of secreted AREG and TGFα in a panel of 8 HNSCC cell lines serum-starved for 48 hours (Fig 6C and 6D). Plotting the concentration of each ligand as a function of the combined anti-proliferative activity of KTN3379 with cetuximab showed a significant correlation between both of these variables (Fig 6C and 6D). This trend was also observed when the concentration of each EGFR ligand is plotted against KTN3379-driven inhibition of phospho-AKT (S8 Fig). In conclusion, our data suggest that while necessary, NRG1 positivity alone does not fully predict KTN3379 anti-tumor activity, but its activity was enriched in cell lines that expressed both NRG1 and high levels of AREG or TGFα. Furthermore, the expression of these ligands correlated with greater anti-tumor activity in response to dual ErbB blockade.

### NRG1 expression and high EGFR homodimer levels correlate with KTN3379 anti-tumor activity

Expression of EGFR ligands in EGFR-expressing tumors would be expected to result in receptor homodimerization and its subsequent activation. To investigate whether high levels of activated EGFR can be used as a surrogate for AREG or TGFα expression, we used the VeraTag proximity-based immunoassay to quantitatively measure EGFR homodimer levels (H11D) in FFPE samples from HNSCC cell lines. Quantitative H11D analysis of HNSCC cell lines revealed a correlation between H11D levels and KTN3379 activity as measured by its anti-proliferative activity in combination with cetuximab (R^2^ = 0.48) or by phospho-AKT inhibition (R^2^ = 0.76) (Fig 6E and 6F). Cells with high levels of H11D demonstrated better anti-tumor effects to treatment with KTN3379 than cell lines with lower H11D levels. This result was predicted based on the observed correlation between KTN3379 activity with increasing levels of AREG and TGFα. We observed a similar enrichment for KTN3379 activity in HNSCC xenograft models, as presented in Fig 3. KTN3379 exhibited significant anti-tumor only in models that expressed both NRG1 and high H11D levels (Cal27 and CTG-0434), but did not have significant activity in a NRG1 positive model with low H11D (CTG-0776) or vice versa, in a NRG1-negative model bearing high H11D levels (CTG-0840). In summary, the simultaneous presence of NRG1 and high EGFR homodimers, or NRG1 with high levels of AREG or TGFαmay represent biomarkers to enrich for HNSCC patients to be treated with KTN3379, particularly in combination with cetuximab.

## Discussion

HNSCC is one of the most common cancers and remains an unmet medical need with urgency for new therapies. We found that ErbB3, HER2 and NRG1 expression are particularly widespread in HNSCC, in agreement with other published studies [29,41], and providing a rationale for evaluation of dual ErbB blockage to improve the benefit of cetuximab treatment in this tumor type. RNA expression analysis in HNSCC patient samples showed that the prevalence of NRG1 expression is very high (85%) and nearly half of HNSCC tumors overexpress the ligand. It has been speculated that threshold levels of NRG1, rather than ErbB3 expression, may be more relevant to ErbB3 activation and predict therapeutic effectiveness with ErbB3-targeting antibodies. Phase 2 clinical data published with anti-ErbB3 mAbs evaluated in lung, ovarian, and breast cancer have suggested that NRG1 expression levels may be a potential biomarker to enrich for patients who will respond to therapy [8,18,40,42]. In contrast to the substantial overexpression of EGFR, we found that ErbB3 is rarely overexpressed in HNSCC. We estimate that ErbB3 expression typically ranges from 10^3^−10^4^ receptors/cell using HNSCC cell lines where receptor levels were measured using quantitative flow cytometry (unpublished observations). However, prevalent co-expression of both EGFR and HER2 along with the substantial expression of NRG1 supported the hypothesis that ErbB3 is activated in a substantial number of patients with HNSCC and is an important disease driver. However, targeting ErbB receptors lacking genetic aberrations (mutations or amplification) that are also disease drivers has resulted in only low response rates [1,43]. This study goes on to further the evidence that anti-ErbB therapies provide more meaningful activity when used in combination.

Cetuximab has shown clinical benefit in after treatment with chemotherapy and radiation (http://www.cancer.gov/about-cancer/treatment/drugs/fda-cetuximab). However, EGFR signaling alone may not be sufficient to potently drive tumor growth since cetuximab therapy does not provide benefit to most patients and, when useful, clinical benefit may not be long-lasting [44]. Efforts to uncover other targets or mechanisms that could potentiate the effectiveness of cetuximab for more HNSCC cancer patients have been an area of active research. In this report, we show that dual ErbB blockade through the combination of cetuximab with the anti-ErbB3 antibody KTN3379, resulted in improved anti-tumor activity relative to either agent alone. Analysis of phospho-ERK and phospho-AKT revealed that cetuximab-mediated EGFR inhibition mainly inhibited the ERK proliferative pathway whereas ErbB3 inhibition robustly inhibits AKT signaling. This finding is consistent with previously published data showing that pharmacological inhibition of EGFR and ErbB3 signaling downregulates ERK and AKT signaling, respectively [45].

It is reasonable to assume that EGFR may be the relevant activating kinase for ErbB3 since ErbB3 has impaired kinase activity, both targets are expressed in HNSCC, and EGFR is known to be a heterodimerization partner for ErbB3 [46,47]. Our data, however, contradict this idea, at least in HNSCC. If ErbB3 activation relies on heterodimer formation with EGFR, then cetuximab should abolish this interaction (by impeding ligand-dependent EGFR/ErbB3 heterodimerization) and result in ErbB3 inhibition. Instead, cetuximab treatment had no effect on ErbB3 phosphorylation in any of the cell lines tested, nor did KTN3379 influence EGFR activity. Rather, treatment with the HER2-blocking mAb pertuzumab resulted in phospho-ErbB3 inhibition, indicating that HER2/ErbB3 heterodimers form preferentially, as described originally by Tzahar et al. [47] and the HER2 kinase primarily activates ErbB3 in HNSCC. Indeed, our analysis of both HNSCC patient tumor samples and cell lines showed that HER2 was robustly expressed in nearly all samples evaluated although overexpression was less common. Our observations are consistent with experiments performed by Li et al [48] who used an exogenous source of NRG1 to activate ErbB3, rather than ligands naturally secreted from tumor cells, as in our case. These data also suggest that HER2-targeting molecules which disrupt ligand-induced HER2-ErbB3 heterodimers may result in clinical efficacy when combined with EGFR-targeting agents. However, these combinations have been poorly tolerated in several clinical trials [49,50], while co-administration of EGFR-targeting agents with anti-ErbB3 mAbs have demonstrated a favorable safety profile.

The high prevalence of NRG1 expression in HNSCC, while necessary for full ErbB3 activation, may be insufficient to fully predict the anti-tumor activity of an ErbB3-blocking antibody and may explain why the clinical studies to date for anti-ErbB3 antibodies have not proven successful [18]. Indeed, several cell lines that expressed both ErbB3 and NRG1 were found not to be sensitive to either KTN3379 and cetuximab on their own or together. We carried a targeted biomarker search around members of the EGFR/ErbB family and found that certain EGFR activation signatures (EGFR homodimers, AREG, or TGFα) in addition to NRG1 expression had a better correlation with KTN3379 activity than NRG1 alone. Analysis of a large panel of HNSCC patient samples demonstrated that NRG1 expression was significantly associated with the EGFR ligands TGFα and AREG, but not the remaining five EGFR ligands (EGF, betacellulin, HB-EGF, epiregulin, and epigen). In contrast, this association was not found in colorectal cancer, representing another solid tumor type where cetuximab and other EGFR-targeting agents have shown clinical benefit and are approved for therapeutic use. Furthermore, HNSCC cell lines that responded to KTN3379 secreted higher levels of both TGFα and AREG relative to either weakly responsive or unresponsive cell lines. This finding was further elaborated measuring EGFR homodimerization (H11D) as an independent signature for EGFR activation and is a surrogate for ligand receptor engagement. HNSCC models expressing both NRG1 and high H11D levels demonstrated an anti-tumor response to treatment with KTN3379 *in vitro*, and this translated to significant single agent KTN3379 activity in cell line-derived and patient-derived HNSCC tumor xenografts in mice with the same biomarker profile (S9 Fig). Although our data support using either NRG1 positivity with either H11D or TGFα/AREG as predictive biomarkers for patient enrichment (with expression cut points to be defined in the clinic), the ability to multiplex NRG1 with TGFα and AREG RNA may be more clinically feasible. Given the targeted nature of this biomarker discovery approach, the data do not exclude the possibility that other biomarkers of response to ErbB3-targeting antibodies exist. Similarly, it will be of interest to evaluate the utility of these and other biomarkers in tumors with acquired resistance to cetuximab.

In conclusion, our preclinical data in HNSCC provide both a unique view of EGFR, HER2, and ErbB3 signaling and a mechanistic rationale to combine EGFR and ErbB3-targeting therapies for the treatment of this tumor type. KTN3379, with its unique epitope and mechanism of action, is particularly suited to this dual blockade and emerging data in clinical trials is supporting further investigation in HNSCC, including a durable complete response in a HNSCC patient treated with KTN3379 and cetuximab [51]. Lastly, our current understanding linking NRG1 expression along with other EGFR activation biomarkers will help guide the future development of anti-ErbB3 mAbs such as KTN3379 in combination with anti-EGFR mAbs such as cetuximab in HNSCC and other tumor types where both EGFR and ErbB3 signaling play a role together.

## Supporting information

S1 Fig NRG expression is higher in HPV-negative and PI3K wild-type HNSCC tumors. NRG RNA levels were measured in HPV- vs. HPV+ and PI3K wild type vs. PI3K mutated human patient HNSCC samples.
(PDF)

S2 Fig EGFR, HER2 and ErbB3 expression is widespread in HSNCC cell lines. **(A)** Flow cytometry data showing expression of EGFR, HER2, and ErbB3 in HNSCC cell lines. ErbB3 was expressed in 7 of 8 cell lines. **(B)** ErbB3 cell surface expression levels in HSNCC cells correlated with ErbB3 levels measured using a H3T VeraTag^®^ assay
(PDF)

S3 Fig EGFR is highly overexpressed in HNSCC, whereas ErbB2 and ErbB3 overexpression is infrequent. TCGA analysis of ErbB receptor expression in HNSCC patient tumor samples. Overexpression is defined using the same criteria as NRG1 (>4-fold above the mean target expression across all tumor types).
(PDF)

S4 Fig Independent or simultaneous inhibition of EGFR or ErbB3 with cetuximab or KTN3379, respectively, demonstrated that EGFR primarily activated the ERK pathway (phospho-ERK), while ErbB3 primarily activated the PI3K/AKT pathway (phospho-AKT) (upper panels). In all assays shown in S4 Fig, antibodies were added at 100 nM for 2 hours to cells grown in reduced serum and where no exogenous ligands were added. In addition, cetuximab had no effect on ErbB3 phosphorylation, indicating that EGFR may not be the activating kinase for ErbB3, and KTN3379, as expected, completely abolished ErbB3 activation but did not affect EGFR activation.
(PDF)

S5 Fig AKT and ErbB3 phosphorylation are pharmacodynamic markers of KTN3379 activity. KTN3379-mediated inhibition of AKT phosphorylation in serum-containing HNSCC cells correlated with KTN3379 anti-proliferative activity when given in combination with cetuximab (top panel). Similarly, phospho-AKT inhibition correlated with inhibition of ErbB3 phosphorylation by KTN3379. ErbB3 phosphorylation was measured using a phospho-ErbB3 VeraTag immunoassay, and the data are presented as the ratio of phospho-ErbB3 in control-treated samples compared to KTN3379-treated samples.
(PDF)

S6 Fig Biomarker expression in HNSCC cell lines. Levels of ErbB receptors, ErbB homodimers (H11D), NRG1, and secreted EGFR ligands TGFα and AREG are shown.
* ErbB receptor and H11D expression levels were measured by VeraTag.
** ErbB3 levels were measured using flow cytometry, and values represent fold ErbB3 expression over a control.
*** NRG1 mRNA levels were measured by QISH, and values represent NRG1 expression over a control.
(PDF)

S7 Fig Association between NRG1 and AREG or TGFα is observed in HNSCC but not in CRC. Significance is defined using a R^2^ cut-off value of 0.25.
(PDF)

S8 Fig High levels of secreted AREG and TGFα are associated with KTN3379 activity in HNSCC cell lines. Levels of secreted AREG and TGFα from a panel of 8 serum-starved HNSCC cell lines were measured after 48 hours. Ligand levels (pg/mL) are plotted as a function of KTN3379-dependent phospho-AKT inhibition, with R^2^ values of 0.57 and 0.52 for AREG and TGFα, respectively.
(PDF)

S9 Fig The ARRIVE guidelines checklist. (PDF)
