# Supplementary material for: ErbB activation signatures as potential biomarkers for anti-ErbB3 treatment in HNSCC
Source: PLoS One. 2017 Jul 19;12(7):e0181356. doi: 10.1371/journal.pone.0181356 (PMC5517012; doi:10.1371/journal.pone.0181356)
Supplement: S1 Fig — NRG RNA levels were measured in HPV- vs. HPV+ and PI3K wild type vs. PI3K mutated human patient HNSCC samples. (PDF) [file pone.0181356.s001.pdf]

	HPV-	HPV+	PI3K wt	PI3K mut
**Count**	84	25	258	45
**Median NRG expression**	9.004	7.453	7.886	8.751
**Outliers**	4	0	9	0
**P-value**	4.95E-8		0.0013	

Figure S1
