# Supplementary figures and images for: ErbB activation signatures as potential biomarkers for anti-ErbB3 treatment in HNSCC
Source: PLoS One. 2017 Jul 19;12(7):e0181356. doi: 10.1371/journal.pone.0181356 (PMC5517012; doi:10.1371/journal.pone.0181356)

**A**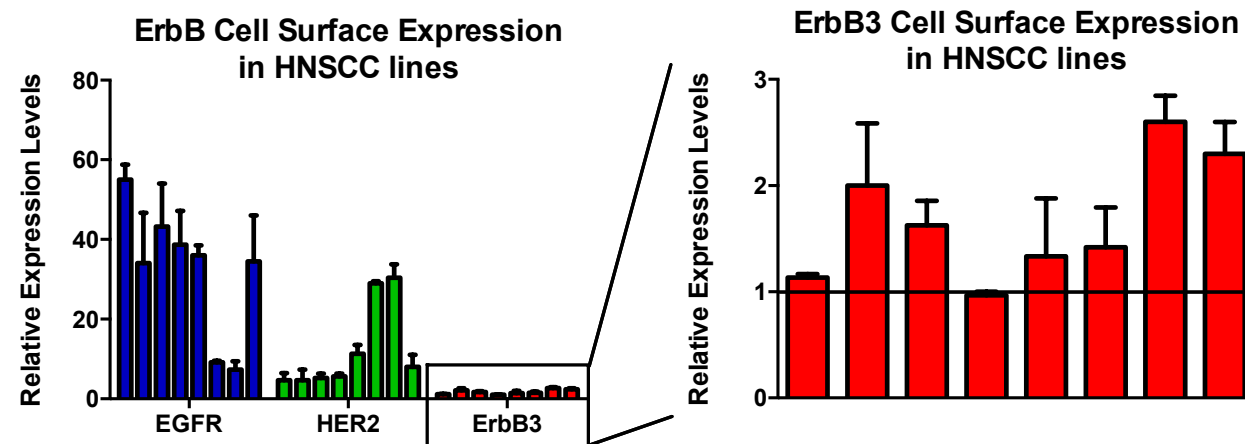**B**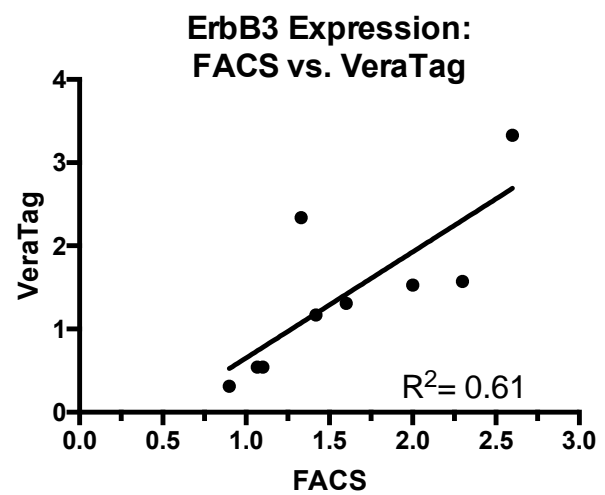

Figure S2

Supplement: S2 Fig — (A) Flow cytometry data showing expression of EGFR, HER2, and ErbB3 in HNSCC cell lines. ErbB3 was expressed in 7 of 8 cell lines. (B) ErbB3 cell surface expression levels in HSNCC cells correlated with ErbB3 levels measured using a H3T VeraTag® assay (PDF) [file pone.0181356.s002.pdf]

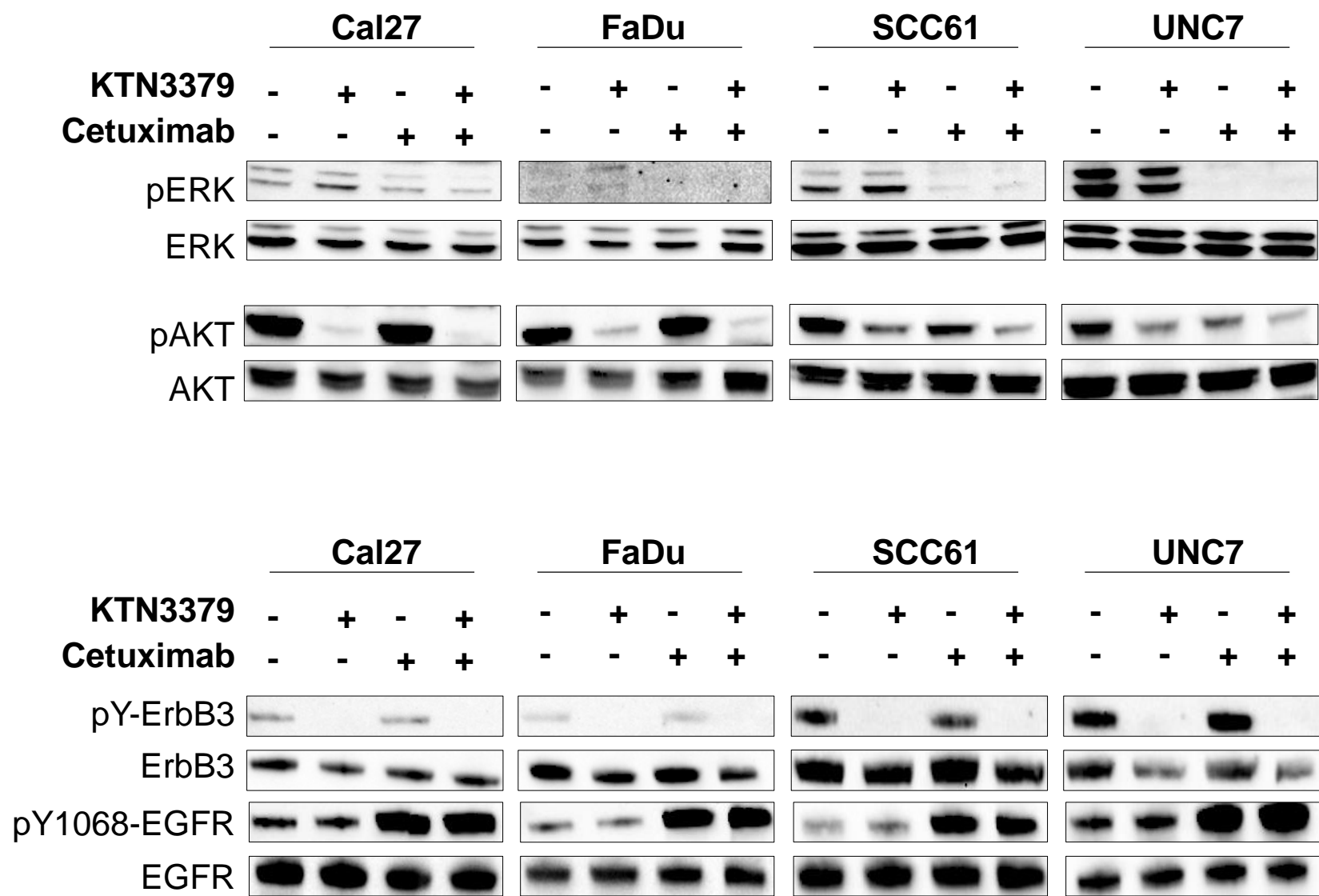

Figure S4

Supplement: S4 Fig — In all assays shown in S4 Fig, antibodies were added at 100 nM for 2 hours to cells grown in reduced serum and where no exogenous ligands were added. In addition, cetuximab had no effect on ErbB3 phosphorylation, indicating that EGFR may not be the activating kinase for ErbB3, and KTN3379, as expected, completely abolished ErbB3 activation but did not affect EGFR activation. (PDF) [file pone.0181356.s004.pdf]

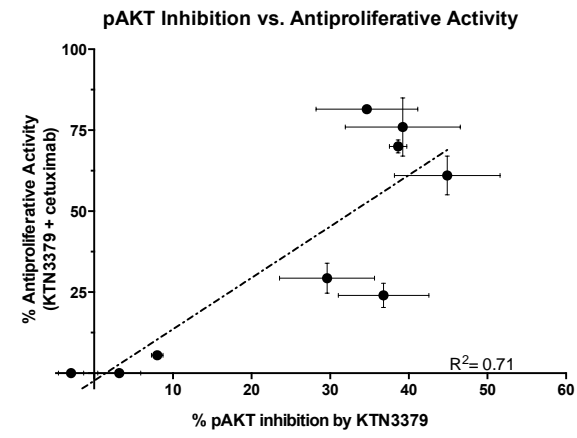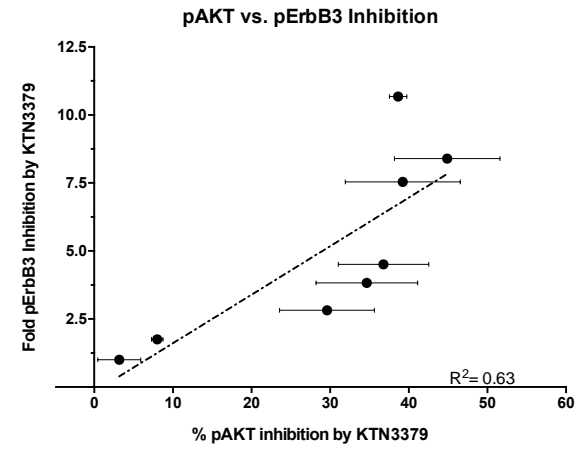

**Figure S5**

Supplement: S5 Fig — KTN3379-mediated inhibition of AKT phosphorylation in serum-containing HNSCC cells correlated with KTN3379 anti-proliferative activity when given in combination with cetuximab (top panel). Similarly, phospho-AKT inhibition correlated with inhibition of ErbB3 phosphorylation by KTN3379. ErbB3 phosphorylation was measured using a phospho-ErbB3 VeraTag immunoassay, and the data are presented as the ratio of phospho-ErbB3 in control-treated samples compared to KTN3379-treated samples. (PDF) [file pone.0181356.s005.pdf]

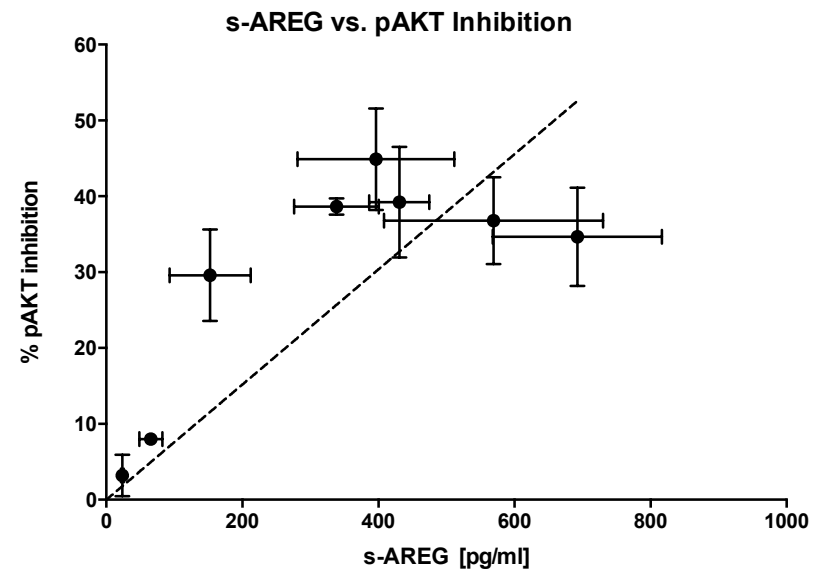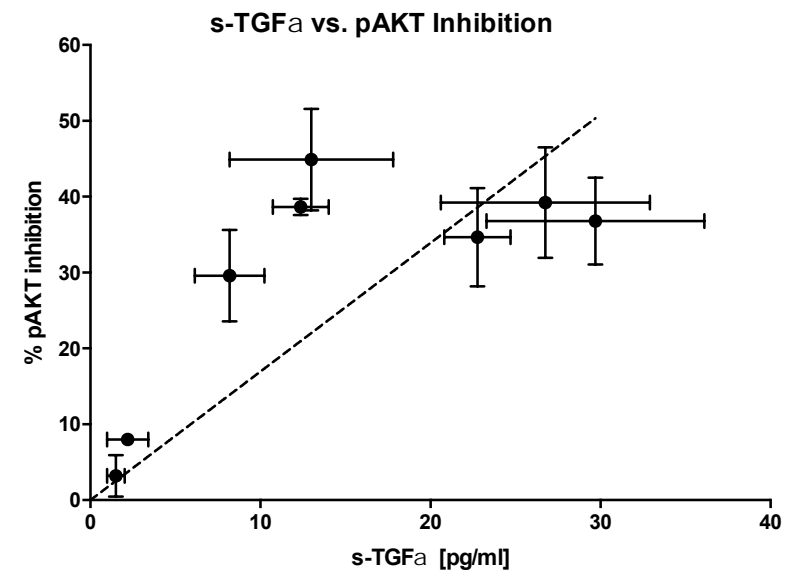

Figure S8

Supplement: S8 Fig — Levels of secreted AREG and TGFα from a panel of 8 serum-starved HNSCC cell lines were measured after 48 hours. Ligand levels (pg/mL) are plotted as a function of KTN3379-dependent phospho-AKT inhibition, with R2 values of 0.57 and 0.52 for AREG and TGFα, respectively. (PDF) [file pone.0181356.s008.pdf]
