# Supplementary material for: ErbB activation signatures as potential biomarkers for anti-ErbB3 treatment in HNSCC
Source: PLoS One. 2017 Jul 19;12(7):e0181356. doi: 10.1371/journal.pone.0181356 (PMC5517012; doi:10.1371/journal.pone.0181356)
Supplement: S3 Fig — TCGA analysis of ErbB receptor expression in HNSCC patient tumor samples. Overexpression is defined using the same criteria as NRG1 (>4-fold above the mean target expression across all tumor types). (PDF) [file pone.0181356.s003.pdf]

Receptor	HNSCC Patient Tumor Samples % overexpression (# samples)
**EGFR**	57.1 (282/494)
**HER2**	8.5 (42/494)
**ErbB3**	2.2 (11/494)

Figure S3
