# Supplementary material for: ErbB activation signatures as potential biomarkers for anti-ErbB3 treatment in HNSCC
Source: PLoS One. 2017 Jul 19;12(7):e0181356. doi: 10.1371/journal.pone.0181356 (PMC5517012; doi:10.1371/journal.pone.0181356)
Supplement: S6 Fig — Levels of ErbB receptors, ErbB homodimers (H11D), NRG1, and secreted EGFR ligands TGFα and AREG are shown. * ErbB receptor and H11D expression levels were measured by VeraTag. ** ErbB3 levels were measured using flow cytometry, and values represent fold ErbB3 expression over a control. *** NRG1 mRNA levels were measured by QISH, and values represent NRG1 expression over a control. (PDF) [file pone.0181356.s006.pdf]

Cell Line	ErbB Levels (VeraTag) *				ErbB3 Levels **	mRNA (QISH)***	EGFR Ligand Secretion (pg/ml)	
	H1T	H2T	H3T	H11D	(FACS)	NRG1	TGFα	AREG
**SCC61**	132.9	27.5	1.5	1882	2.00	1.76	12.4 ± 1.65	338 ± 62.2
**UNC10**	55.9	19.6	0.3	32	0.97	1.88	1.5 ± 0.52	23.6 ± 2.11
**UNC7**	95.6	12.8	1.3	581	1.62	2.78	13 ± 4.8	396 ± 115
**SCC35**	106.7	19.0	0.5	889	1.13	3.60	8.19 ± 2.05	153 ± 59.5
**Cal27**	112.1	24.2	1.6	1559	2.30	2.34	26.7 ± 6.15	431 ± 44.1
**FaDu**	99.7	28.8	2.3	226	1.33	1.86	22.8 ± 1.96	692 ± 124
**SCC9**	49.2	50.4	1.2	33	1.42	1.99	2.19 ± 1.21	65.4 ± 17.0
**Detroit562**	81.7	29.7	3.3	651	2.60	3.06	29.7 ± 6.40	569 ± 161

Figure S6
