# Supplementary material for: ErbB activation signatures as potential biomarkers for anti-ErbB3 treatment in HNSCC
Source: PLoS One. 2017 Jul 19;12(7):e0181356. doi: 10.1371/journal.pone.0181356 (PMC5517012; doi:10.1371/journal.pone.0181356)
Supplement: S7 Fig — Significance is defined using a R2 cut-off value of 0.25. (PDF) [file pone.0181356.s007.pdf]

EGFR Ligand	HNSCC (n=303)	CRC (n=537)
**AREG**	P=8.53 E-28, R^2=0.33	P=6.26 E-7, R^2=0.04
**TGFα**	P=2.87 E-20, R^2=0.25	P=1.77 E-5, R^2=0.03
**EGF**	P=0.319, R^2=0.003	P=0.161, R^2=0.004
**EREG**	P=7.21 E-17, R^2=0.21	P=3.28 E-13, R^2=0.09
**HB-EGF**	P=1.85 E-6, R^2=0.07	P=2.32 E-4, R^2=0.02
**Epigen**	P=8.37 E-11, R^2=0.13	P=0.742, R^2=0.00
**BTC**	P=1.79 E-4, R^2=0.05	P=0.186, R^2=0.003

Figure S7
